# IViT: An Incremental Learning Method for Object Detection of Hidden Hazards in Transmission Line Corridors

**DOI:** 10.3390/s26010158

**Published:** 2025-12-25

**Authors:** Min Li, Kun Fan, Peng Luo, Junping Liu

**Affiliations:** 1 School of Computer Science and Artificial Intelligence, Wuhan Textile University, Wuhan 430200, China; 2008031@wtu.edu.cn (M.L.); jpliu@wtu.edu.cn (J.L.); 2 School of Electrical and Automation, Wuhan University, Wuhan 430072, China; thornluo@whu.edu.cn

**Keywords:** UAV inspection, incremental learning, object detection, knowledge distillation

## Abstract

The inspection of power transmission lines using unmanned aerial vehicles primarily relies on object detection. However, the continuous emergence of new obstacle types necessitates frequent updates to detection models, leading to substantial retraining costs. To address this challenge, we propose a novel framework named IViT, which integrates incremental learning with a hybrid CNN-Transformer architecture for improved identification. We combined knowledge distillation with the elastic response selection distillation strategy to enhance detection performance for old classes and strengthen knowledge retention through star convolutional residual blocks constructed via element-wise multiplication. We designed a separable convolution aggregation block that integrates PConv with an attention mechanism, effectively merging global and local information to improve detection accuracy. Finally, we unified the two modules into a hybrid block. In the static detection task, IViT achieves a mAP of 55.3%, a mAP_50_ of 83.6%, and a mAP_75_ of 61.0%. For the incremental detection task, it attains a mAP of 57.8%, a mAP_50_ of 79.7%, and a mAP_75_ of 62.3%. Extensive experiments on the transmission line corridor external damage dataset and the INSPLAD dataset demonstrate that IViT exhibits outstanding detection performance compared to mainstream static object detection models and incremental object detection models.

## 1. Introduction

The transmission line corridor refers to the strip-shaped area beneath high-voltage power lines extending a specified width on both sides. Monitoring and inspecting transmission lines is one of the important responsibilities to ensure the safe operation of the transmission network. Transmission lines are susceptible to various potential hazards, which may result in short-term or long-term power outages [1]. Therefore, efficient and reliable monitoring of potential threats in the transmission line corridor area is crucial for ensuring the safe operation of the transmission network. Transmission line inspection uses computer vision to identify and locate power transmission lines and their surrounding objects. This technology leverages images captured by UAVs, surveillance cameras, and other devices, and utilizes deep learning algorithms such as convolutional neural networks (CNN) and visual transformer (ViT) [2] to achieve automatic detection of towers, buildings, vehicles, and other potential obstacles. At present, commonly used inspection methods for transmission lines include manual inspection, robot inspection, helicopter inspection, and UAV inspection [3].

In recent years, with the advancement of computing hardware and the establishment of large-scale annotated image datasets, deep learning–based computer vision algorithms have achieved remarkable success in object detection tasks [4]. Mao et al. [5] proposed an automated defect detection system for dual-inline package (DIP) in an industrial context, utilizing a ConSinGAN generative adversarial network to construct the training dataset. This system integrates the YOLOv7 deep learning model to achieve intelligent recognition of surface defects and pin defects. Chou et al. [6] proposed an automated defect detection method for metal sheets based on ConSinGAN data augmentation with YOLOv9, achieving high-precision, real-time defect detection. This method has been successfully integrated into manufacturing hardware and SCADA systems. In the field of UAV inspection, Liu et al. [7] designed the Mask R-CNN algorithm for detecting objects in close-range images, while utilizing edge detection and Hough transform techniques for distant images to accomplish UAV-based inspection. Zhang et al. [8] introduced multi-scale feature alignment and a multi-scale consistency regularization module, which effectively enhanced the accuracy of transmission line inspection models. Liang et al. [9], based on a self-constructed multi-defect dataset, adopted Faster R-CNN with transfer learning to detect transmission line defects under complex backgrounds and lighting conditions. Furthermore, Li et al. [10] improved the YOLOX object detection network through lightweight design, integrating coordinate attention and the α-DIOU loss function, thereby enabling precise target localization and real-time attitude adjustment during high-altitude UAV autonomous inspection—mitigating object deviation. Huang et al. [11] optimized the YOLOv5 architecture by incorporating attention mechanisms, improving both detection accuracy and efficiency in transmission line inspection. Additionally, Miao et al. [12] developed a two-stage fine-tuning strategy based on the SSD framework, achieving efficient and accurate detection of multiple types of insulators in aerial images with complex backgrounds.

Although the aforementioned methods have achieved notable progress in improving the accuracy of object detection for power transmission line inspection tasks, these studies generally fail to account for the increasing diversity in the types of electrical infrastructure and surrounding objects. Consequently, UAV-based inspection systems often require complete model retraining when encountering new data, leading to high computational costs and complex model update procedures. To address this issue, this study introduces the concept of incremental learning into the power transmission line inspection task. By enabling UAVs to recognize new data without retraining the model jointly with all previous data, the proposed approach significantly reduces computational overhead and simplifies model updates, thereby enhancing the efficiency and flexibility of UAV inspections. Incremental learning allows models to acquire new knowledge or adapt to new tasks without full retraining. By updating the model using previously learned representations, it avoids the extensive time and computational resources required for full retraining. This paradigm is particularly well-suited to the dynamic environments of power transmission line inspection. Defect datasets suffer from class imbalance, subtle inter-class differences, and cluttered backgrounds. Under these conditions, CNNs capture local patterns but miss long-range dependencies, while Transformers model global relationships but often lose fine-grained details. Therefore, integrating CNN and Transformer components allows the model to simultaneously leverage local structure and global context, providing a more robust representation for complex visual scenes. The main contributions of this paper are summarized as follows:

- We constructed an incremental object detection framework called IViT. This framework uses a distillation-based incremental learning method, enabling the model to learn new classes without retraining the entire network. By adopting a hybrid CNN–Transformer architecture, the model captures both fine-grained local features and global dependencies, thus achieving more accurate feature representations in hazard detection.
- We proposed an efficient Star-shaped Convolution Residual Block (SCRB). By employing deep convolution to stabilize the features of old classes and utilizing element-wise multiplication to construct high-dimensional feature interactions, the model enhances its feature retention capability. In incremental hazard detection, this approach effectively mitigates the model’s forgetting of old hazards.
- We proposed a Separable Convolution Aggregation Block (SCAB) that integrates local convolution operations with attention mechanisms to enhance the model. This approach improves the model’s capacity to capture global contextual information within images, thereby providing greater adaptability when dealing with complex hazard images.

## 2. Related Work

In the field of object detection, Two-Stage detection algorithms, such as the R-CNN series, Faster R-CNN, and Mask R-CNN, demonstrate excellent performance in detection accuracy. However, their complex network structures and multi-stage inference processes result in slower detection speeds, making it challenging to meet real-time detection requirements. In contrast, One-Stage detection algorithms like SSD and RetinaNet demonstrate significant advantages in detection speed by directly predicting object locations and classes, yet their accuracy in detecting small objects and within complex backgrounds still requires improvement. Among the various object detection algorithms, the YOLO (You Only Look Once) series has emerged as a research hotspot for industrial defect detection due to its unique single-stage detection architecture and outstanding real-time performance. YOLOv9 [13] introduces Programmable Gradient Information (PGI) and Generalized ELAN (GELAN) technologies to address the issue of information loss in deep network training through programmable gradient path planning, while optimizing the multi-scale feature fusion mechanism. YOLOv10 [14] focuses on improving the training strategy by implementing a more refined loss function design and adaptive data augmentation techniques, significantly enhancing inference speed while maintaining detection accuracy. YOLOv11 [15] further reconstructs the network architecture by incorporating an improved CSPNet structure and BiFPN feature fusion mechanism, adopting an anchor-free detection paradigm and integrating model pruning and quantization-aware training techniques to optimize accuracy, speed, and model complexity.

Incremental learning is a machine learning approach that enables a model to acquire new knowledge from novel data or tasks while mitigating the forgetting of previously learned information. A central challenge in this paradigm is catastrophic forgetting, where learning new tasks leads to the degradation of prior knowledge [16]. To address this issue, two commonly adopted strategies are regularization-based and replay-based methods. Regularization methods impose constraints on the loss function of new tasks to prevent new knowledge from overwriting the old, typically without requiring access to original data. Representative techniques include EwC [17] and EBLL [18]. In contrast, replay methods retain old knowledge by utilizing stored samples or synthesized features from previous tasks during training, with notable examples such as ER [19] and DGR [20]. Another solution involves the integration of knowledge distillation. In incremental learning, knowledge distillation facilitates the transfer of learned knowledge from an old model to a new one, thereby alleviating catastrophic forgetting. LwF [21] pioneered the use of knowledge distillation in this context by incorporating predictions from the old model as soft targets and introducing a distillation loss term into the objective function, followed by fine-tuning on new tasks. iCaRL [22] follows a similar philosophy to LwF but relaxes the constraint of not using old data, allowing the model to better preserve feature representations of earlier tasks.

Incremental Object Detection (IOD) is a technique that applies incremental learning to the visual domain. Compared to incremental classification, IOD presents greater complexity due to the need to localize and classify objects simultaneously [23] first adapted the concept of LwF to the Fast R-CNN detector, pioneering the integration of knowledge distillation in this context. Subsequent research has further explored the application of incremental learning principles to object detection. For instance, RILOD [24] introduced a real-time dataset system capable of continuously collecting training images with automated annotation of both categories and bounding boxes, facilitating efficient learning of new classes. SID [25] enhanced this approach by selective distillation at strategic layers with relational instance knowledge. Meanwhile, ONCE [26] extended the framework to few-shot incremental object detection, addressing the challenge of learning new categories with limited data.

Distillation-based methods represent a typical memory-replay-free incremental paradigm. Our proposed IViT follows this distillation-based IOD framework, where distillation constraints enable the model to preserve stable knowledge without relying on historical images or pseudo samples. For hazard-inspection datasets that exhibit long-tailed distributions, this strategy prevents tail classes with limited samples from being overwhelmed by frequently occurring head classes during incremental learning, thereby effectively mitigating the combined challenges of class imbalance and catastrophic forgetting.

## 3. Methodology

This section begins with an overview of the proposed framework, followed by a detailed presentation of the introduced Star Convolution Residual Block (SCRB) and Separate Convolution Aggregation Block (SCAB).

### 3.1. Overview of the Proposed Method

**Figure 1:**
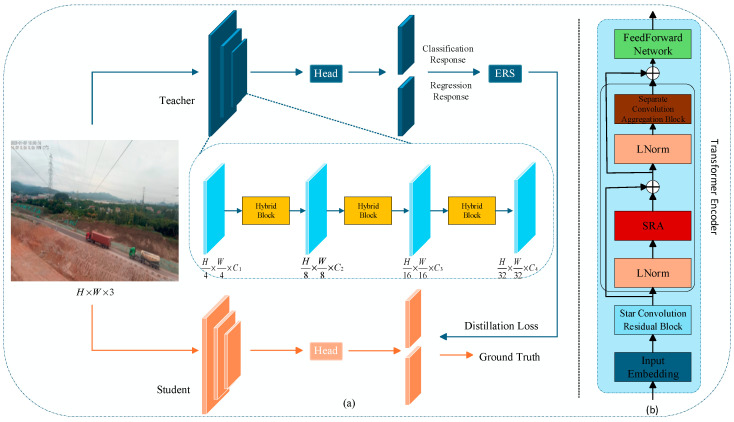
Overview of the IViT framework. (**a**) The overall framework of IViT comprises a student model and a teacher model. (**b**) Detailed illustration of the hybrid block, which mainly consisting of Star Convolution Residual Block and Transformer Encoder.

The framework of the proposed method is illustrated in Figure 1a. It achieves incremental object detection through knowledge distillation, employing an identical network architecture for both the student and teacher models. A key component is the novel hybrid block, which integrates CNN and Transformer into a unified framework, as depicted in Figure 1b.

During the incremental training process, the model trained in the previous stage is first fully frozen at the beginning of the new stage and used as the teacher model, with its weights kept unchanged throughout the entire stage to ensure the stability of previously learned knowledge representations. The student model is then initialized with the same network architecture as the teacher. During training, the input image is first forwarded through the teacher model to obtain high-level semantic and localization responses. Subsequently, the student model processes the same input to learn the newly introduced categories of the current stage, while being optimized with the elastic response selection (ERS) distillation strategy [27], which captures potential positional information through regression responses and analyzes statistical characteristics of different responses to automatically select appropriate distillation nodes. Candidate responses are first scored based on their confidence values, and the mean and standard deviation are computed to dynamically define an elastic threshold. Responses exceeding this threshold are selected as distillation nodes, ensuring the retention of high-quality classification information and the most representative localization outputs. This ensures that the student detector extracts the most valuable responses, thereby enhancing the effectiveness of knowledge transfer.

Specifically, ERS automatically selects the most informative distillation nodes based on the statistical characteristics of the teacher model’s regression responses, enabling the student model to obtain more effective positional guidance at critical locations. Meanwhile, incremental localization distillation allows the student model to learn the inherent ambiguity in positional information, which helps maintain the stability of bounding box regression during incremental updates. The parameters of the student model are continuously updated through classification loss, regression loss, and incremental distillation loss. Ultimately, this framework achieves accurate prediction of annotated ground truth while fulfilling the objectives of incremental detection. The overall loss function *L_total_* is defined as:

(1) $$L_{total}=L_{model}+L_{ers\_cls}+L_{ers\_bbox}$$

where *L_model_* represents the detector’s classification and localization loss, which is utilized to train the student detector for detecting novel objects. *L_ers_cls_* denotes the incremental classification loss, while *L_ers_bbox_* corresponds to the incremental regression loss. Its pseudo algorithm is shown in Algorithm 1.

In the model architecture, the input image is processed by a three-stage encoder, where each stage is responsible for extracting features at different hierarchical levels. Throughout this process, the spatial dimensions of the feature maps are progressively reduced to {1/2, 1/4, 1/8, 1/16} of the original image size. This reduction enhances the feature abstraction, enabling the model to capture characteristic information at varying scales. Regarding the design of the encoder, given an input image *I* ∈ R*^H^^×W^^×C^*, where H, W, and C denote the height, width, and number of channels of the image, respectively, the input first passes through an embedding layer:

(2) $$X_{embed}=InputEmb(I)$$

The image data is converted into a feature representation suitable for subsequent processing. Then, it passes through a Star Convolution Residual Block:

(3) $$X_{dc}=SCRB(X_{embed})$$

**Figure 2:**
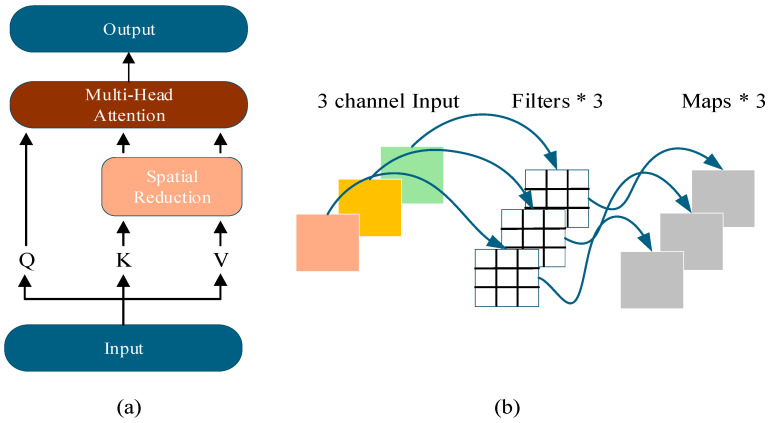
The Structure of Spatial Reduction Attention and Depthwise Convolution. (**a**) Spatial Reduction Attention. (**b**) Depthwise Convolution.

The primary objective of this step is to mitigate catastrophic forgetting via deep convolutional operations, while simultaneously reducing the number of model parameters. The feature maps pass through two residual connection blocks. The first residual block applies normalization, followed by Spatial Reduction Attention (SRA) [28]. The structure of SRA is illustrated in Figure 2a. reducing the spatial dimensions of the keys (K) and values (V), SRA decreases the number of elements required for similarity computation, lowering computational cost:

(4) $$SRA(Q,K,V)=Concat({head}_{0},...,{head}_{N_{i}})W^{0}$$

(5) $${head}_{j}=Attention(QW_{j}^{Q},SR(K)W_{j}^{K},SR(V)W_{j}^{V})$$

(6) $${\;X}_{res1}=X_{dc}+SRA(Norm(X_{dc}))$$

where $W_{j}^{Q}\in R^{C_{i}\times d_{head}},W_{j}^{K}\in R^{C_{i}\times d_{head}}{,W}_{j}^{V}\in R^{C_{i}\times d_{head}}$ and $W^{O}\in R^{C_{i}\times C_{i}}$ are linear projection parameters, N_i_ denotes the number of attention heads in the i stage, and SR(·) is an operation that reduces the spatial dimensions of the input sequence. The entire process enhances training stability through normalization and strengthens the model’s feature extraction ability via the attention mechanism. The second residual connection block also starts with normalization, followed by the Separate Convolution Aggregation Block:

(7) $$X_{res2}=X_{res1}+SCAB(Norm(X_{res1}))$$

This process enhances model performance and computational efficiency through partial channel convolution during feature fusion. Finally, the feature maps are processed by a Feed Forward Network layer:

(8) $$Y=FeedForwardNetwork(X_{res2})$$

This layer adopts a series of linear transformations and nonlinear activation functions to further refine features and enhance the model’s decision-making capability.
**Algorithm 1:** Incremental trainInput:           Pre-trained teacher model T; Student model S;           Old class dataset $D_{old}$; New class dataset $D_{new}$;           Number of incremental iterations N. Output:           Updated student model  S with knowledge of old and new classes. 1. Student-teacher initialization        Freeze all parameters of teacher model T.        Initialize student model S with the same architecture as T.        Define total loss using Equation (1); 2. Incremental training iterations        For each mini-batch from $D_{new}$:                Forward pass through teacher model T to extract high-level semantic and localization responses.                 Forward pass through student model S to predict new classes. 3. ERS-based distillation updates                Score teacher regression responses and select high-quality responses as distillation nodes using elastic thresholds.                Compute incremental classification and regression losses ($L_{ers\_cls},\;L_{ers\_bbox}$).                Update student model S using combined loss $L_{total}$. 4. parameter freezing and model output.                 Optionally freeze parts of student model S to retain old class knowledge.                 Output the updated student model S.

### 3.2. Star Convolution Residual Block

**Figure 3:**
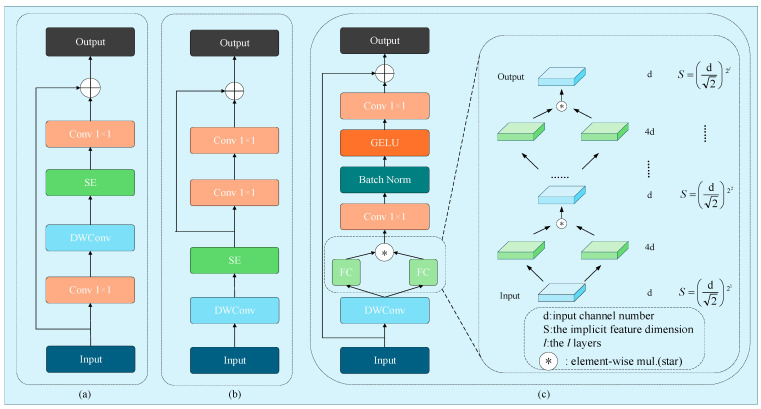
Three structures of Depthwise Convolution. (**a**) is a MobileNet block. (**b**) is a RepViT block. (**c**) is a designed Star Convolution Residual Block.

DWConv was originally proposed by MobileNet [29], primarily serving to substantially reduce model parameters and enhance computational efficiency. Its structure is depicted in Figure 2b. DWConv employs a set of convolutional kernels with a channel count of 1, each processing a single input channel, with the number of channels equal to the number of kernels. This design enables independent convolutional operations per channel, facilitating more effective reuse of existing feature representations. In incremental object detection tasks, such feature reuse allows the model to more efficiently retain knowledge from previous tasks when learning new ones. RepViT [30] also introduces improvements to the modules in MobileNet, effectively reducing the model parameters, with their structures illustrated in Figure 3a,b. The SE layer enables adaptive recalibration of channel-wise feature responses, allowing the model to emphasize features beneficial for the current task, thereby effectively improving model accuracy without significantly increasing computational complexity. However, during incremental learning, when the model learns data from new classes, the parameters of these SE layers are updated to optimize recognition of the new classes, which leads to a sharp decline in the model’s feature representation capacity for old classes. To address this issue, Star Convolution Residual Block (SCRB) is proposed, as shown in Figure 3c. SCRB enhances the retention of old knowledge by leveraging the independent channel convolutions of DWConv alongside the implicit high-dimensional feature representation capability of the star operation.

Inspired by StarNet [31], the star operation can implicitly map input features into an extremely high-dimensional nonlinear feature space, an effect analogous to the kernel trick in Support Vector Machines (SVMs), but without practically increasing the network width. This allows the network to perform computations in a low-dimensional space while leveraging the powerful representational capacity of high-dimensional features. The star operation is defined as:

(9) $$\;w_{1}^{T}x\;*\;w_{2}^{T}x=\;\left(\sum_{i=1}^{d+1}w_{1}^{i}x^{i}\right)⋇\left(\sum_{j=1}^{d+1}w_{2}^{j}x^{j}\right)=\sum_{i=1}^{d+1}\sum_{j=1}^{d+1}w_{1}^{i}w_{2}^{j}x^{i}x^{j}$$

(10) $${=\alpha }_{\left(1,1\right)}x^{1}x^{1}+...+\alpha_{\left(4,5\right)}x^{4}x^{5}+...+\alpha_{\left(d+1,d+1\right)}x^{d+1}x^{d+1}$$

(11) $$\alpha_{\left(i,\;j\right)}=\left\{\begin{array}{c} {\;\;\;\;\;\;\;\;\;\;w}_{1}^{i}w_{2}^{j}\;\;\;\;\;\;\;\;\;if\;i==j,\\ w_{1}^{i}w_{2}^{j}+w_{1}^{i}w_{2}^{j}\;\;\;if\;i\;!=j. \end{array}\right.\;$$

where $w=\left[\begin{array}{c} W\\ B \end{array}\right]$, and similarly, $x=\left[\begin{array}{c} X\\ 1 \end{array}\right]$. w_1_, w_2_, x ∈ R^(*d*+1)×1^, *d* is the input channel number. Use *i*, *j* to index channels, where α is the coefficient for each term.we can expand it into a composition of $\frac{\left(d+2)(d+1\right)}{2}$ distinct items, yet we achieve a representation in a $\frac{\left(d+2)(d+1\right)}{2}\approx {\left(\frac{d}{\sqrt{2}}\right)}^{2}$ implicit dimensional feature space. This is in a single-layer neural network, and when extended to multiple layers, it can be expressed as:

(12) $$O_{1}\;=\;\sum_{i=1}^{d+1}\sum_{j=1}^{d+1}w_{\left(1,\;1\right)}^{i}w_{\left(1,\;2\right)}^{j}x^{i}x^{j}\;\;\;\;\;\;\;\;\;\;\;\;\;\;\;\;\;\;\;\;\;\;\;\in R^{{\left(\frac{d}{\surd 2}\right)}^{2^{1}}}$$

(13) $$\;\;\;\;\;\;\;\;\;\;O_{2}=W_{2,1}^{T}O_{1}\;*\;\;\;W_{2,2}^{T}O_{1}\;\;\;\;\;\;\;\;\;\;\;\;\;\;\;\;\;\;\;\;\;\;\;\;\;\;\;\;\;\in R^{{\left(\frac{d}{\surd 2}\right)}^{2^{2}}}$$

(14) $$\;\;\;\;\;\;\;\;O_{l}=W_{l,1}^{T}O_{l-1}\;*\;\;\;W_{l,2}^{T}O_{l-1}\;\;\;\;\;\;\;\;\;\;\;\;\;\;\;\;\;\;\;\;\;\;\;\;\;\in R^{{\left(\frac{d}{\surd 2}\right)}^{2^{l}}}$$

where *O*_i_ denote the output of *l*-th star operation. The Star operation, when stacked across multiple layers, can significantly and exponentially expand the latent feature dimensions. By performing element-wise interactions between different linear transformations of the input, it generates high-dimensional, non-linear feature representations that capture subtle distinctions between classes. This enhanced feature granularity enables the model to more effectively preserve knowledge of previously learned classes.

We utilize the channel-independent characteristics of DWConv to stabilize the representations of old class features and integrate it with the star operation to achieve higher-dimensional feature representations without an explicit increase in network width. Specifically, the input feature map first undergoes a DWConv to reduce model parameters. The resulting features are then split into two parts, where subspace features are fused via the star operation and further extracted through a convolutional layer, followed by batch normalization enhancing training speed and stability. A GELU activation function is applied, and the activated features are processed by another convolutional layer. Finally, a skip connection is established with the original input. The entire process can be formulated as follows:

(15) $$F_{1},\;F_{2}=DWConv(F_{in})$$

(16) $$F_{3}=Star(F_{1},F_{2})$$

(17) $$F_{4}={Conv}_{1\times 1}(F_{3})$$

(18) $$F_{5}=BatchNorm(F_{4})$$

(19) $$F_{6}=GELU(F_{5})$$

(20) $$F_{7}={Conv}_{1x1}(F_{6})$$

(21) $$F_{out}=F_{in}+F_{7}$$

### 3.3. Separate Convolution Aggregation Block

**Figure 4:**
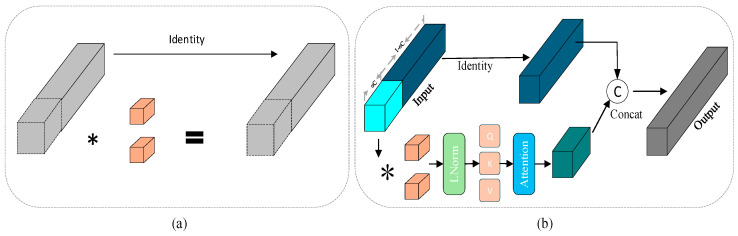
The structure of Partial Convolution and Separate Convolution Aggregation Block. (**a**) Partial Convolution. (**b**) Separate Convolution Aggregation Block. The asterisk represents Convolution.

To address the trade-off between accuracy and efficiency in existing neural networks, particularly the high memory access costs incurred when increasing network width to enhance representational capacity. [32] proposed an efficient alternative: Partial Convolution (PConv). The structure of PConv is illustrated in Figure 4a. While traditional full convolution uniformly applies convolutional kernels across all input channels, resulting in high computational redundancy and frequent access to large-scale input feature maps and convolution weights, especially under high-resolution input scenarios, making deployment on resource-constrained devices challenging—PConv innovatively applies convolution only to a subset of input channels while bypassing the remaining channels. This approach significantly reduces both computational complexity and memory access frequency per layer. However, PConv has limited receptive fields, limiting its ability to capture global feature representations. In incremental object detection tasks, this limitation can lead to difficulties in distinguishing between old and new categories, thereby degrading model performance.

The attention mechanism effectively captures long-range dependencies and enhances model’s expressiveness. We integrate PConv with an attention mechanism to construct a Separated Convolutional Aggregation Block (SCAB). It leverages an attention mechanism to capture long-range dependencies across channels and spatial positions, effectively compensating for the limited receptive field of PConv networks and thereby enhancing global semantic modeling. This enables the model to better distinguish between old and new knowledge. Such a synergistic effect not only strengthens the model’s feature representation capability but also reduces redundant computations, achieving an improved balance between performance and efficiency. Specifically, the original input channels are split into multiple subgroups, with convolution applied to a portion of them. Attention mechanisms are then incorporated to improve accuracy, enabling the model to focus on learning specific features while preserving the integrity of other critical information in the original channels. The structure is illustrated in Figure 4b. This module balances local perception with global modeling capabilities, significantly improving both efficiency and expressiveness in computationally constrained scenarios while effectively reducing redundant computations and memory access. Specifically, the input *X* ∈ R*^H^^×^^W^^×^^C^* is first divided into two parts, *X*_1_ and *X*_2_:

(22) $$X_{1},X_{2}=Split(X_{int},\left|C_{\alpha },C-C_{\alpha }\right|)$$

where Cₐ = αC (with α = 1/4). This choice is primarily motivated by a trade-off between computational efficiency and feature representation capacity. Such channel splitting preserves feature diversity while significantly reducing redundant computations. Here, *X*_1_ and *X*_2_ represent the partitioned segments of the original input. Subsequently, *X*_2_ is passed through a convolutional layer for feature refinement:

(23) $$X_{3}=Conv(X_{2})$$

the input *X*_3_, which represents the features refined by the convolutional layer, undergoes normalization through LayerNorm to stabilize the feature distribution and facilitate subsequent processing:

(24) $$X_{4}=LayerNorm(X_{3})$$

*X*_4_ denotes the normalized features, which are then processed by a self-attention mechanism to adaptively recalibrate feature importance, thereby enhancing model performance:

(25) $$X_{5}=Attention(X_{4})$$

*X*_5_ represents the output obtained after processing *X*_4_ through the attention mechanism. Subsequently, *X*_5_ is concatenated with the unprocessed original input *X*_1_:

(26) $$X_{out}=Concat(X_{1},X_{5})$$

*X_out_* is the final output obtained after concatenation.

## 4. Experiments

In this section, we evaluated the proposed method on two power datasets. We first introduce the dataset, select evaluation metrics, and describe the experimental details. Next, we conducted comparative experiments on several advanced models. In addition, we also conducted ablation studies to further validate the effectiveness of our method. Finally, we conducted visualization experiments to more intuitively perceive the differences between various methods.

### 4.1. Datasets

- Transmission Line Corridor External Damage Dataset: A dataset comprising ten categories of external damage objects was collected, including cranes, tower cranes, excavators, transmission towers, dump trucks, forklifts, concrete mixers, concrete pump trucks, pile drivers, and gas cylinders. The dataset was expanded to 9611 images through image augmentation techniques such as scaling and random cropping.
- InsPLAD Dataset [33]: The dataset comprises 18,000 images of electrical components, covering seventeen categories including insulators. From these, fourteen categories that do not overlap with our custom dataset were selected for experimentation. This selection was made to prevent experimental bias that could arise from having identical classes present in both the old and new task datasets.

The dataset was partitioned into training and testing sets using a 9:1 ratio. The Transmission Corridor External Damage dataset was employed as the old classes, while the InsPLAD dataset was introduced as the new classes to validate the proposed method.

### 4.2. Implementation Details and Evaluation Metrics

- Implementation Details: The experiments in this paper are deployed on mmdetection [34]. The learning rate is set at 0.02, momentum is at 0.9, using the stochastic gradient descent algorithm, with a batch size of 1, on two RTX 2080Ti GPU.
- Evaluation Metrics: We use Mean Average Precision (mAP) as evaluation metrics.

### 4.3. Comparative Experiments

To validate the proposed method, comparative experiments were first conducted on our self-constructed Transmission Corridor External Damage dataset against leading static object detection methods—including ATSS [35], RTMDet [36], TOOD [37], VFNet [38], YOLOv9 [13], YOLOv10 [14], YOLOv11 [15], and RT-DETR [39]—to evaluate its detection performance. For fairness, we train these methods from scratch. Subsequently, the self-built dataset was integrated with the large-scale public dataset InsPLAD, treating the former as old classes and the latter as new classes, and comparisons were made with state-of-the-art incremental object detection methods: IOD [40], MLIFSOD [41], ERD [27], and CLDETR [42]. This integrated dataset approach provides a comprehensive assessment of the model’s generalization capacity and detection accuracy. The comparative results are summarized in Table 1, Table 2 and Table 3.

#### 4.3.1. Results of Comparative Experiments

Table 1 presents a comparative analysis between IViT and several static object detection methods. The detection performance varies notably across the evaluated models. Among the compared methods, RTMDet demonstrates the lowest performance, with mAP, mAP_50_, and mAP_75_ values of only 46.0%, 72.3%, and 50.2%. The mAP and mAP_75_ of TOOD reaches a maximum of 54.9% and 60.5%. YOLOv9 achieves the highest mAP_50_ at 84.6%. The proposed IViT surpassed TOOD in both mAP and mAP_75_, reaching values of 55.3% and 61.0%, while achieving a mAP of 83.6%, which is second only to YOLOv9. Although IViT has more parameters than it, its computational cost is much higher, reaching 254 GFLOPs. These results indicate that our method achieves state-of-the-art detection performance, enabling more accurate identification of potential hazards in complex transmission corridor external damage scenarios. This competitive advantage can be attributed to the well-designed architecture of the IViT framework.

**Table 1:** Quantitative comparisons with SOTA Static Object Detection Methods.(The bold numbers in the table represent the best performance data in this indicator).

Method	mAP (%)	mAP_50_ (%)	mAP_75_ (%)	Params (M)	FLOPS (G)
ATSS [35]	53.7	79.5	59.5	32.134	200
RTMDet [36]	46.0	72.3	50.2	52.262	79
TOOD [37]	54.9	79.5	60.5	32.039	196
VFNet [38]	53.6	80.4	58.3	32.730	188
YOLOv9 [13]	54.8	**84.6**	59.2	2.554	103
YOLOv10 [14]	48.2	75.1	49.1	2.980	82
YOLOv11 [15]	48.0	74.9	52.3	2.591	65
RT-DETR [39]	52.5	79.8	57.1	42.781	130.5
IViT	**55.3**	83.6	**61.0**	32.949	254

Table 2 presents a performance comparison between IViT and SOTA incremental object detection methods prior to the incremental learning phase. Among the benchmark methods, IOD demonstrates the lowest performance, with mAP, mAP_50_, and mAP_75_ values of only 42.3%, 70.7%, and 43.4%. CLDETR achieved the highest results with scores of 55.2%, 81.3%, and 60.8% across the respective metrics. In comparison, the proposed IViT surpasses CLDETR on all evaluation metrics. Although IViT achieves only marginal improvements on mAP and mAP_75_, it reaches a mAP_50_ of 83.6% and simultaneously attains GFLOPs comparable to CLDETR while using only 32.949 M parameters. This indicates that, compared with other incremental object detection methods, IViT is markedly more effective at detecting the presence of targets. These results demonstrate that IViT attains SOTA detection performance compared against both static and incremental object detection baselines.

**Table 2:** Quantitative comparisons with SOTA incremental Object Detection Methods.

Method	mAP (%)	mAP_50_ (%)	mAP_75_ (%)	Params (M)	Flops (G)
IOD [40]	42.3 ± 0.4	70.7 ± 0.4	43.4 ± 0.4	38.912	108
MLIFSOD [41]	52.8 ± 0.3	76.4 ± 0.5	53.2 ± 0.4	30.983	187
ERD [27]	54.9 ± 0.3	80.7 ± 0.4	60.5 ± 0.4	32.279	206
CLDETR [42]	55.2 ± 0.2	81.3 ± 0.4	60.8 ± 0.3	34.376	279
IViT	**55.3** ± 0.3	**83.6** ± 0.4	**61.0** ± 0.4	32.949	254

Table 3 presents the comparative performance between IViT and leading incremental object detection methods after the incremental learning phase. Specifically, IViT achieves 40.1%, 65.7%, and 44.2% on old classes and 71.7%, 90.2%, and 76.4% on new classes across the respective metrics, demonstrating significant superiority over all other methods in all evaluation criteria. IOD demonstrates outstanding performance in detecting new categories, with its mAP_50_ value surpassing all other methods. However, it shows significantly inferior results in detecting old categories, revealing its limitations in mitigating catastrophic forgetting. Both MLIFSOD and ERD achieve notable improvements in old class detection, but this comes at the expense of compromised new class detection capability. CLDETR exhibits competitive overall performance, maintaining satisfactory results on old categories while achieving reasonable detection capability on new ones. However, IViT outperforms CLDETR on both old and new classes. Specifically, for old classes, which achieves a mAP50 of 65.7%, exceeding CLDETR by 3.4%, and for new classes, which reaches a mAP50 of 90.2%, outperforming CLDETR by 3.8%. These results demonstrate that IViT can effectively distinguish between old and new categories while preserving strong detection performance, further validating the superiority of our approach in balancing the retention of old knowledge with the acquisition of new categories. In summary, IViT not only reaches the detection level of state-of-the-art methods but also effectively alleviates catastrophic forgetting through its integrated CNN-Transformer architecture. By simultaneously maintaining old class knowledge while adapting to new classes, our method demonstrates exceptional performance in incremental object detection.

**Table 3:** Quantitative comparisons with SOTA incremental Object Detection Methods after increment.

Method	The Top 10 Categories			The Last 14 Categories			mAP (%)	mAP_50_ (%)	mAP_75_ (%)
	mAP (%)	mAP_50_ (%)	mAP_75_ (%)	mAP (%)	mAP_50_ %)	mAP_75_ (%)			
IOD [40]	21.4 ± 0.4	38.2 ± 0.4	21.9 ± 0.4	59.7 ± 0.3	87.2 ± 0.3	67.3 ± 0.3	43.7 ± 0.4	66.8 ± 0.4	48.4 ± 0.4
MLIFSOD [41]	28.5 ± 0.3	46.6 ± 0.4	30.4 ± 0.3	63.8 ± 0.3	84.7 ± 0.4	70.1 ± 0.3	47.9 ± 0.3	67.5 ± 0.4	52.7 ± 0.3
ERD [27]	38.1 ± 0.4	59.7 ± 0.4	42.3 ± 0.4	64.7 ± 0.3	81.0 ± 0.4	71.4 ± 0.4	53.6 ± 0.3	72.2 ± 0.4	59.3 ± 0.4
CLDETR [42]	39.6 ± 0.4	62.3 ± 0.4	43.5 ± 0.4	69.5 ± 0.2	86.4 ± 0.4	72.6 ± 0.3	56.2 ± 0.2	74.6 ± 0.3	60.1 ± 0.3
IViT	**40.1** ± **0.3**	**65.7** ± **0.3**	**44.2** ± **0.4**	**71.7** ± **0.3**	**90.2** ± **0.3**	**76.4** ± **0.3**	**57.8** ± **0.3**	**79.7** ± **0.3**	**62.3** ± **0.3**

#### 4.3.2. Visualization Results

**Figure 5:**
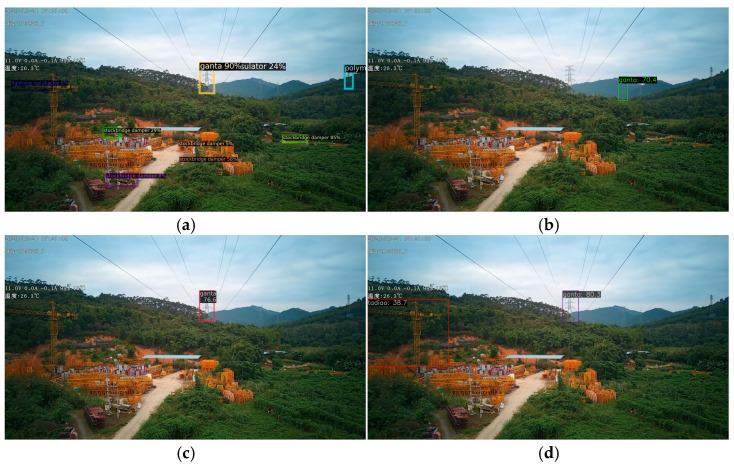
Comparison of old classes recognition with advanced incremental detection methods. (**a**) IOD. Incorrect detection. (**b**) MLIFSOD.Missed detection. (**c**) ERD. Missed detection. (**d**) CLDETR. Correct detection. (Incorrect detection indicates detection error, Missed detection indicates detection omission, and Correct detection indicates detection is completely correct).

**Figure 6:**
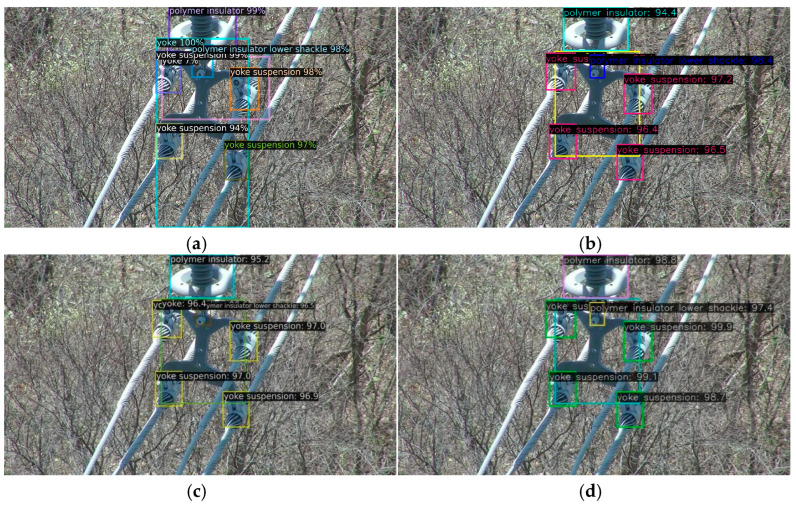
Comparison of new classes recognition with advanced incremental detection methods. (**a**) IOD. (**b**) MLIFSOD. (**c**) ERD. (**d**) CLDETR.

**Figure 7:**
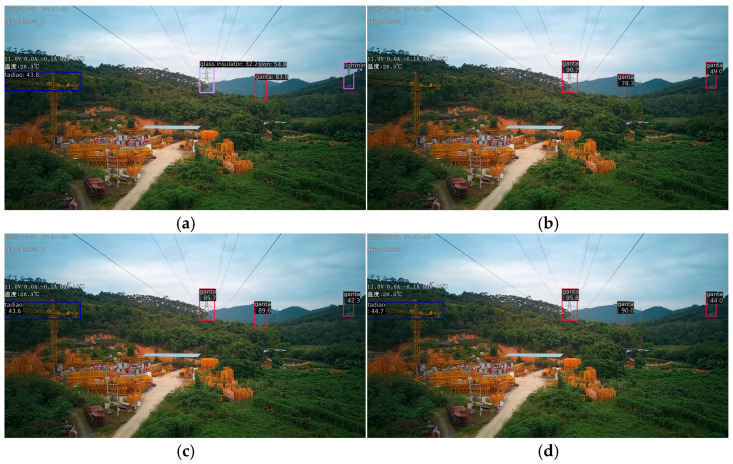
The ablation result of the old classes after increment. (**a**) Transformer Encoder. Incorrect detection. (**b**) Elastic Response Selection. Missed detection. (**c**) Star Convolution Residual Block. Correct detection. (**d**) Separate Convolution Aggregation Block. Correct detection.

**Figure 8:**
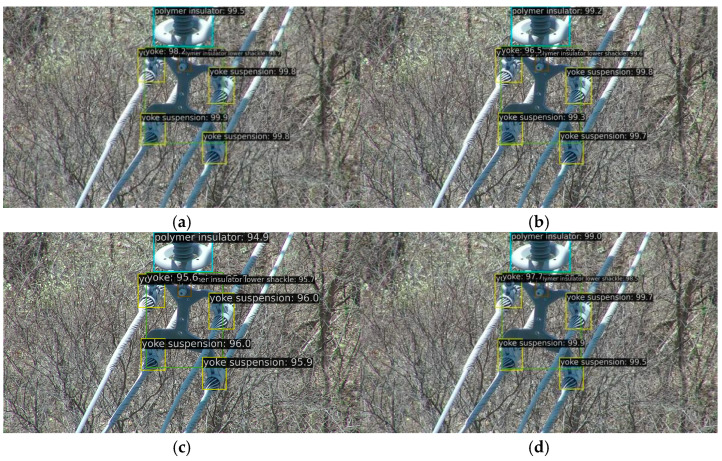
The ablation result of the new classes after increment. (**a**) Transformer Encoder. (**b**) Elastic Response Selection. (**c**) Star Convolution Residual Block. (**d**) Separate Convolution Aggregation Block.

Figure 5, Figure 6 and Figure 7d, and 8d present qualitative comparisons between IViT and leading incremental object detection methods. Among them, Figure 5 and Figure 7d illustrate detection results on old classes. It can be clearly observed that IOD performs the poorest—while it generates multiple bounding boxes, the category predictions are entirely incorrect and numerous duplicate detections are present. IOD exhibits insufficient semantic representation and redundant bounding-box localization in complex background scenarios. In contrast, MLIFSOD and ERD exhibit notable deficiencies in class discriminability and feature representation, detecting only a single category on our dataset. Moreover, they suffer from severe missed detections, indicating limited robustness in complex background scenarios. CLDETR demonstrates relatively strong overall performance and is capable of recognizing multiple categories, its feature modeling capacity remains insufficient, resulting in missed detections. This suggests that its fine-grained feature extraction and relational modeling in complex scenarios are still inadequate. IViT accurately and completely identifies all categories present in the images, fully demonstrating its advantage in mitigating catastrophic forgetting. Figure 6 and Figure 8d present the detection results on new classes. Among the compared methods, IOD remains the least effective, while CLDETR achieves the best performance. Notably, IViT further improves upon CLDETR, successfully preserving detection capability for new classes while maintaining strong performance on old ones. These visual comparisons validate the comprehensive superiority of our proposed method in detecting both old and new categories.

### 4.4. Ablation Experiments

To validate the effectiveness of the proposed modules, ablation studies were conducted on the Transmission Corridor External Damage dataset and the large-scale public dataset InsPLAD. The detection performance was first evaluated on the proprietary dataset, followed by incremental learning experiments that integrated both datasets to enhance the model’s generalization capability. Leveraging the global modeling capacity of Transformers, the transformer-based GFLv1 was selected as the baseline method, into which the ERS distillation strategy, SCRB, and SCAB were sequentially incorporated.

Quantitative results are presented in Table 4 and Table 5. As shown in Table 4, prior to incremental learning, the baseline transformer-based model achieved mAP, mAP_50_, and mAP_75_ scores of 54.4%, 82.7%, and 58.3%, respectively. After integrating the SCRB, these metrics improved to 54.5%, 83.3%, and 59.9%, demonstrating the module’s contribution to enhancing detection performance. The introduction of SCAB led to more pronounced improvements, with mAP, mAP_50_, and mAP_75_ reaching 55.3%, 83.6%, and 61.0%, further validating the efficacy of the proposed design in boosting detection accuracy.

Table 5 summarizes the ablation results after incremental learning. Without any specific strategy, the model achieved 33.5% mAP, 57.4% mAP_50_, and 35.3% mAP_75_ on old classes, while attaining 72.8% mAP, 89.8% mAP_50_, and 78.9% mAP_75_ on new classes. Upon incorporating the ERS strategy, the performance on old classes increased by 5.4%, 6.2%, and 6.2%, respectively, while results for new classes remained stable at 72.0%, 89.6%, and 78.0%. This indicates that the ERS strategy effectively mitigates the catastrophic forgetting problem while having minimal impact on the detection of new categories. To further preserve knowledge of old classes, the SCRB was introduced, leading to additional minor gains of 0.2%, 1.3%, and 0.3% on old classes. However, this module slightly reduced performance on new classes, with decreases of 2.6%, 1.1%, and 3.4% observed, suggesting a trade-off between retaining old knowledge and adapting to new classes. To restore the model’s capability in recognizing new classes, the SCAB was designed to enhance overall detection performance. With the incorporation of SCAB, the model not only maintained its improved performance on old classes but also achieved significant gains in overall detection accuracy. Specifically, the detection performance for old classes improved to 40.1% mAP, 65.7% mAP_50_, and 44.2% mAP_75_, while performance for new classes remained at high levels of 71.7% mAP, 90.2% mAP_50_, and 76.4% mAP75. Experimental results and analysis demonstrate that the introduced ERS strategy, along with the proposed SCRB and SCAB, each plays a distinct role in successfully mitigating catastrophic forgetting and effectively preserving detection capability for new categories. Figure 7 and Figure 8 present the visualization results of the ablation study. As shown in Figure 7, without the ERS strategy, the model misclassifies some old-class objects as new categories. After incorporating the ERS strategy, the detection results become more stable with improved categorical discrimination, though some missed detections persist and the overall precision remains limited. The introduction of the SCRB further enhances the model’s recognition capability for old classes, significantly mitigating the catastrophic forgetting problem. The overall detection performance of the model is further enhanced by the integration of the SCAB. Figure 8 illustrates that while introducing the ERS strategy and SCRB initially reduces detection performance for new classes, the final integration of SCAB effectively compensates for this decline and enhances new-class detection. Overall, the proposed IViT framework successfully maintains old-class knowledge and mitigates catastrophic forgetting, while simultaneously achieving competitive detection performance on new classes. These results validate the method’s exceptional detection capability and incremental learning effectiveness in power line inspection tasks.

## 5. Conclusions

This paper introduces IViT, an incremental object detection framework that systematically integrates knowledge distillation, convolutional neural networks, and Transformer architectures. Compared with ERD and CL-DETR, IViT adopts a hybrid architecture that balances local feature efficiency and global relational modeling, providing a more efficient optimization foundation for incremental learning. In contrast to conventional hybrid CNN-Transformer detectors, IViT, as an incremental detector, employs a flexible knowledge distillation mechanism that enables the retention of recognition capability for old classes while progressively learning new ones. The proposed framework incorporates an Elastic Response Selection strategy for distillation, which effectively preserves knowledge of previously learned classes. Through synergistic interaction between the Star Convolutional Residual Block (SCRB) and the Separate Convolution Aggregation Block (SCAB), the model demonstrates enhanced capability in recognizing both old and new categories. The deep fusion of CNN and Transformer components enables robust feature representation learning. This also introduces certain memory and computational overheads. SCRB leverages depthwise convolutions and element-wise multiplication to strengthen the retention of old-class features; however, the construction of high-dimensional feature spaces results in a slight increase in computational cost when multiple layers are stacked. SCAB effectively improves global semantic modeling by integrating local convolutions with attention mechanisms, yet the additional matrix operations introduced by the attention module may impose burdens on resource-constrained deployment settings.

Although IViT achieves compelling performance, it still possesses certain limitations. Under complex industrial conditions, motion blur distorts feature extraction and blurs boundary information, which in turn increases feature confusion between new and old tasks, ultimately exacerbating catastrophic forgetting. Occlusion leads to the loss of critical target features, limiting the model’s generalization ability and leading to accumulated localization errors. High-frequency UAV vibration introduces image distortion and geometric deformation, undermining the stability of feature representations. Furthermore, we discuss the practical applicability of the proposed method in UAV-based inspection scenarios. Since the framework does not rely on memory replay, incremental updates introduce no additional inference latency, enabling the system to meet the near-real-time requirements of UAV operations. Under challenging conditions such as camera shake, rapid viewpoint changes, and weather-induced noise, the CNN components maintain stable local texture and edge representations, while the Transformer’s global modeling capability helps preserve semantic consistency under occlusion and geometric variations. In addition, the SCRB enhances the stability of old-class feature representations, and the SCAB strengthens global contextual aggregation, collectively improving the model’s robustness to environmental disturbances. For practical deployment, onboard computational capacity and power consumption must be taken into account to ensure that the model operates reliably within limited compute and battery constraints while maintaining real-time detection performance.

In future work, cross-domain incremental learning may be further explored to accommodate distribution shifts under varying conditions, enabling the model to maintain stable performance as new scenes and emerging hazard types are continuously introduced. Meanwhile, multi-task extensions could be incorporated to jointly perform small-object detection, component segmentation, and hazard classification within a unified framework, thereby enhancing the overall scene understanding in complex power-line inspection environments. Additionally, a temporal consistency module can be integrated to exploit information from consecutive UAV video frames, suppressing jitter-induced noise and short-term false detections, and consequently improving the model’s robustness in real-world inspection scenarios.

## Figures and Tables

**Table 4:** Ablation experiment results before incremental learning.

Method	mAP (%)	mAP_50_ (%)	mAP_75_ (%)
Transformer Encoder	54.4	82.7	58.3
SCRB	54.5	83.3	59.9
SCAB	**55.3**	**83.6**	**61.0**

**Table 5:** Ablation experiment results after incremental learning.

Method	The Top 10 Categories			The Last 14 Categories			mAP (%)	mAP_50_ (%)	mAP_75_ (%)
	mAP (%)	mAP_50_ (%)	mAP_75_ (%)	mAP (%)	mAP_50_ (%)	mAP_75_ (%)			
Transformer Encoder	33.5	57.4	35.3	72.8	89.8	78.9	55.7	77.2	59.1
ERS	38.9	63.6	41.5	72.0	89.6	78.0	57.6	79.1	61.8
SCRB	39.1	64.9	41.8	69.4	88.5	74.6	57.2	79.2	61.6
SCAB	**40.1**	**65.7**	**44.2**	71.7	**90.2**	76.4	**57.8**	**79.7**	**62.3**

## Data Availability

The links to the InsPLAD Dataset used in the paper are https://github.com/andreluizbvs/InsPLAD (accessed on 9 May 2025).If you are interested in the Transmission Line Corridor External Damage Dataset used in this paper, please contacting the corresponding author if necessary.
